# Estrogen Receptor-Low Positive (ER-Low) Breast Cancer: A Unique Clinical and Pathological Entity

**DOI:** 10.3390/curroncol33020122

**Published:** 2026-02-18

**Authors:** Gavino Faa, Eleonora Lai, Pina Ziranu, Andrea Pretta, Ekta Tiwari, Mariele Dessì, Cinzia Solinas, Giorgio Saba, Francesco Loi, Claudia Codipietro, Simona Graziano, Laura Ottelio, Massimo Dessena, Ferdinando Coghe, Jasjit S. Suri, Luca Saba, Mario Scartozzi

**Affiliations:** 1 Department of Medical Sciences and Public Health, University of Cagliari, AOU Cagliari, 09124 Cagliari, Italy; gavinofaa@gmail.com; 2 Department of Biology, College of Science and Technology, Temple University, Philadelphia, PA 19122, USA; 3 Medical Oncology Unit, University Hospital and University of Cagliari, 09124 Cagliari, Italy; pi.ziranu@gmail.com (P.Z.); an.pretta@gmail.com (A.P.); marieledessi@tiscali.it (M.D.); czsolinas@gmail.com (C.S.); sabagiorgio@live.it (G.S.); francescoloi@hotmail.it (F.L.); claudiacodipietro96@gmail.com (C.C.); s.graziano@aoucagliari.it (S.G.); l.ottelio@studenti.unica.it (L.O.); marioscartozzi@gmail.com (M.S.); 4 Department of Innovation, Global Biomedical Technologies, Inc., Roseville, CA 95661, USA; etiwari@calpoly.edu; 5 S.S. Senologia Chirurgica, Chirurgia Polispecialistica, Policlinico Universitario di Monserrato, Azienda Ospedaliera Universitaria, 09124 Cagliari, Italy; mades64@yahoo.it; 6 Clinical-Microbiological Laboratory, University Hospital of Cagliari, 09042 Cagliari, Italy; fcoghe@aoucagliari.it; 7 Stroke Monitoring and Diagnostic Division, Atheropoint™, Roseville, CA 95661, USA; jasjit.suri@atheropoint.com; 8 Department of Radiology, Azienda Ospedaliera Universitaria of Cagliari, 09124 Cagliari, Italy; lucasabamd@gmail.com

**Keywords:** breast cancer, estrogen receptor-low (ER-low) positive, breast cancer pathology, artificial intelligence, endocrine treatment, chemo-immunotherapy

## Abstract

ER-low breast cancer (1–9% ER expression) represents a biologically and clinically distinct entity at the interface between ER-positive and ER-negative disease. Although traditionally managed as hormone receptor-positive, mounting evidence indicates that ER-low tumors share molecular signatures, aggressive behavior, and chemotherapeutic responsiveness with triple-negative breast cancer. Accurate ER assessment is hindered by methodological variability and interpretative challenges, leading to potential misclassification and suboptimal treatment choices. While the benefit of endocrine therapy remains uncertain, ER-low tumors consistently show sensitivity to chemotherapy and promising responses to neoadjuvant chemo-immunotherapy, paralleling outcomes observed in triple-negative breast cancer cohorts. Emerging artificial intelligence tools, including digital pathology and multimodal deep learning, may enhance ER quantification, reduce observer variability, and enable more precise patient stratification. This review synthesizes current pathological and clinical insights into ER-low breast cancer and highlights evolving therapeutic strategies, with a forward-looking perspective on AI-driven approaches to optimize personalized treatment for this challenging subtype.

## Simple Summary

Estrogen receptor-low positive (ER-low) breast cancer, defined by 1–9% ER expression, is increasingly recognized as a distinct subtype with clinical behavior that differs from both classic ER-positive and ER-negative disease. Although historically managed as hormone-responsive, ER-low tumors often display molecular and pathological features closer to triple-negative breast cancer, including a higher grade, increased proliferation, and early relapse risk. These characteristics help explain the limited and inconsistent benefit of endocrine therapy observed in multiple studies. In contrast, ER-low tumors typically show strong responses to chemotherapy and encouraging results with neoadjuvant chemo-immunotherapy, aligning with treatment patterns used for triple-negative disease. Accurate determination of ER levels remains challenging due to technical and interpretive variability, which can influence therapeutic decisions. Emerging artificial intelligence tools in digital pathology may improve the precision of ER quantification and help identify which patients may still benefit from endocrine therapy. This review summarizes current evidence and highlights future research directions.

## 1. Introduction

Breast cancer (BC) represents the most frequent tumor and the first cause of cancer death in women worldwide [1]. Indeed, BC is the most common aggressive cancer in women, with an incidence of 2.3 million globally in 2022, and 670,000 deaths in the same year [1].

BC is a heterogeneous disease and is traditionally classified into different subtypes according to the expression of hormone receptors (estrogen and progesterone), human epidermal growth factor receptor (HER2) and Ki67: luminal (hormone receptor-positive), HER2-positive, triple-negative (TNBC) (when neither hormone receptors nor HER2 are present) [2]. These subtypes show a distinct prognosis and require specific treatment strategies [3]. The luminal subtype accounts for the majority of BC. The estrogen receptor (ER) is highly expressed in 70–75% of BC, whereas the progesterone receptor (PR) is co-expressed in more than 50% of them [2,3,4].

The expression of ERs in BC cells is a relevant prognostic and predictive biomarker. ERs are useful for establishing a therapeutic strategy and especially for endocrine treatment (ET) [5]. ER+ >1% has historically been considered a sufficient condition to assign candidate patients to ET, regardless of the entity of ER expression [6].

ER-low BC is a subtype of BC characterized, using immunohistochemistry (IHC), by the expression of ERs in 1% up to 9% of cancer cells [7].

Recently, ER-low BC has emerged as a potential independent entity, in-between ER+ and ER-negative BC [8,9,10,11,12]. It is a BC subtype characterized, using IHC, by the expression of ERs in 1% up to 9% of cancer cells [7]. ER-low BC is rare; it accounts for 2–7% of all ER+/HER2-negative BC and seems to present a heterogeneous biological behavior. Indeed, recent studies on the immune and gene-expression profile in ER-low and ER-negative BC evidenced strict similarities between the two subtypes, appearing more likely to be basal-like compared to ER-high (>10%) BC, with comparable clinical outcomes and responses to therapy, including immunotherapy. Moreover, evidence regarding the efficacy of ET in this patient subgroup remains controversial, as ER-low patients may derive limited or no benefit from ET [9,13,14,15,16,17,18,19,20,21,22,23,24,25]. Thus, to date, no international standardization of treatment therapeutic guidelines for these patients are available [26].

The definition of this subtype of BC is itself challenging, particularly in distinguishing ER-low tumors, with up to 9% of cells expressing ERs, from ER-positive tumors, in which ≥10% of cancer cells express ERs; for this reason, it appears susceptible to marked interindividual variability among pathologists, justifying the definition of “biology chaos” [11].

In this review, we aim to provide recent evidence on ER-low BC from a clinical and pathological point of view by summarizing the most important pathological and clinical aspects of this peculiar BC subtype, and by underlying the most recent therapeutic strategies for the best management of these patients together with a focus on future perspectives.

## 2. Pathological and Clinical Aspects

### 2.1. Pathological Features

ER expression in cancer cells, determined by IHC, represents a mandatory requirement for all new diagnosed BC. The American Society of Clinical Oncology/College of American Pathologists (ASCO/CAP) recommends that the ER status should be determined in all invasive BC, as well as in all BC recurrences. The ER status of the tumor is considered positive when at least 1% of cancer cell nuclei are immunoreactive for ER antibody in a tissue sample [25].

Patients with BC who express ERs on cancer cells are, in general, considered responsive to ET. On the other hand, patients with cancer cells not expressing ERs in general are not responders [9]. According to the American Society of Clinical Oncology (ASCO) current guidelines (2020), BC with 1–10% of cancer cells expressing ERs should be categorized as ER-low positive [25]. Nonetheless, the same guidelines do not provide a clear statement specifying that ER-low BC represents a different clinical entity from BC with ER expression in >10% of cancer cells and that consequently, its management and treatment are different, thus laying the foundations for controversies and clinical questions [25].

The definition, using histopathology, of ER-low BC represents a challenge for pathologists, and has been defined as a matter of confusion [27]. Being based on a semi-quantitative method, the interpretation of the IHC expression of ERs on cancer cells is affected by a high number of variables that render the semi-quantitative evaluation susceptible to marked variability, particularly in cases of weak immunostaining. The most important variable factors are tissue handling and fixation time, the different antibody clones utilized, the antibody concentration and the detection system utilized. Furthermore, the IHC slide readout and interpretation may undergo marked interindividual variability, according to the different experience of pathologists [28]. All these variables taken together can explain the possible under-, or alternatively, over-estimation of ER expression, which, particularly in cases of low expression levels, may lead to misclassification of the ER status, ending with a suboptimal therapeutic strategy [29]. Generative artificial intelligence (AI) systems could open a new scenario even in the field of BC, toward the goal of a preventive, personalized and precision oncology [30,31].

Recent evidence suggests that ER-low positive BC is a heterogeneous group regarding the molecular profile, prognosis and response to ET [29]. The hypothesis of heterogeneity is underlined by the different response of ER-low BC to ET [9].

### 2.2. Clinical Characteristics

**Figure 1:**
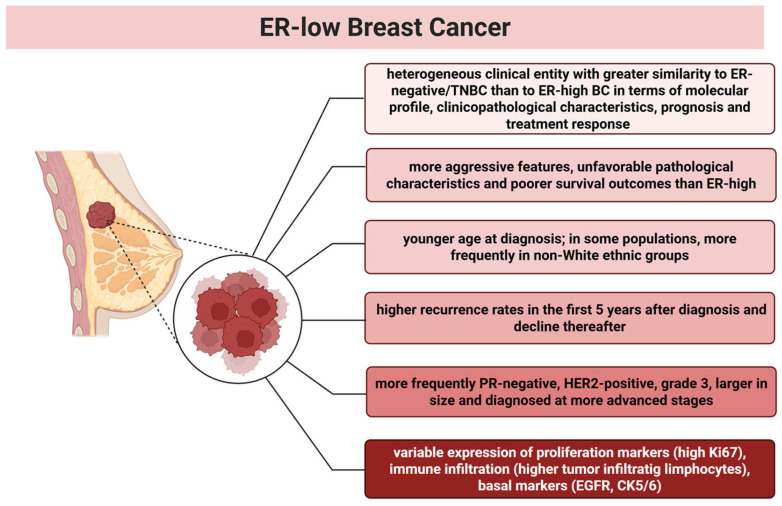
Main clinical and pathological features of ER-low BC. The figure was created with www.biorender.com, and the appropriate license for publication was obtained.

Clinical features of ER-low BC have been assessed in various studies, with some controversial evidence (Figure 1).

Overall, ER-low BC seem to present a similar profile to ER-negative BC in terms of molecular aspects, clinicopathological characteristics, prognosis and treatment response [11]. Indeed, the difference between ER-low and ER-negative appears smaller than the one between ER-low and ER-high BC [11].

Most studies demonstrated that ER-low BC patients have more advanced disease, poor clinicopathologic features and worse survival [27,32].

Luo et al. included in their research 5466 BC patients, of whom 277 (5.1%) were ER-low, 3457 (62.2%) were ER-high, and 1732 (32.7%) were ER-negative. ER-low patients were more likely to have advanced, PR-negative, HER2-positive or grade III BC compared to ER-high patients, suggesting a more aggressive profile [33].

In the study by Massa et al. in 921 early-stage BC patients (I-III), ER-low tumors were more likely to have lobular histology and HER2-0 status compared to ER-negative, whereas no differences in stage, nodal status, grade, or proliferation rate were detected [6].

Poon et al. demonstrated that ER-low BC might be a heterogeneous entity, but in general, they were more likely associated with larger tumor, higher grade, more necrosis, more tumor-infiltrating lymphocytes, higher pathological node stage, high Ki67, HER2, EGFR and CK5/6 positivity but PR and androgen receptors negativity (*p* ≤ 0.039). When compared to ER-negative BC, ER-low showed higher PR expression, lower grade, lower expression of CK5/6 and CK14 (*p* ≤ 0.014). Furthermore, lymph vascular invasion (*p* = 0.024) and younger age (*p* = 0.005) were more frequent when compared to ER-negative BC and survival was worse in the downstaged cases [32].

Another study assessed the comparison of clinical outcomes between ER-low BC and ER-negative BC in a monocenter cohort of non-metastatic ER-low patients receiving (neo)adjuvant chemotherapy. No significant differences in age at BC diagnosis, histologic grade, Ki67 and frequency of special type histology was reported. Conversely, ER-low BC patients were more likely to receive a diagnosis at a more advanced disease stage (stage I 19% versus 39% and stage III 24% vs. 13%, respectively; *p* = 0.031). Thus, ER-low BC showed similar aggressive clinicopathological characteristics and even a higher frequency of locally advanced disease at diagnosis as compared to ER-negative disease [21].

The annual pattern of recurrence in ER-low BC appears comparable to that observed in TNBC and ER-negative disease [11]. Recurrence rates are highest within the first five years after diagnosis (1.5–3.5%) and subsequently decline to 1–3% during years 5–10. This trend contrasts with ER-high BC, which shows relatively lower recurrence rates in the first five years (1.0–2.5%) but a marked increase between years 5 and 10 (2.5–4.0%) [33].

A prospective, multicenter registry of treated BC patients was analyzed to evaluate the impact of ER and PR expression (low versus negative) on clinicopathological features, treatment patterns, and survival outcomes in HER2-negative disease. Within the ER-low subgroup, PR expression ranged from 1% to 3%; 21.5% (14/65) of patients exhibited both ER and PR expression between 1% and 10%, while 52.3% (34/65) showed PR expression below 1%. A total of 26.2% (17/65) were ER-negative with PR expression 1–10%. No statistically significant differences in ethnic distribution, disease stage, lymph node involvement, or germline BRCA1/2 mutations prevalence were identified. These results confirm the key similarities between ER-low BC and TNBC [14].

Yi et al. conducted a retrospective study in nearly 10.000 patients that showed significant differences between ER-low and ER-high BC: ER-low patients were younger (median age 53 versus 56 years), more frequently had a clinical TNM stage II/III (62% vs. 44%), were less frequently of white ethnicity (66% versus 72%), and had ductal carcinoma (84% versus 73%) and grade 3 disease (82% versus 28%) more often. Moreover, they received preoperative chemotherapy (48% versus 29%) less commonly. Conversely, the only significant differences compared to ER-negative BC were a lower proportion of stage II/III clinical TNM staged disease (62% versus 68%) and a higher frequency of a ductal histotype (84% vs. 88%). At a median follow-up of 5.1 years, ER-low BC had worse survival rates than ER+ tumors, with and without adjustment for clinical stage and grade; on the other hand, no significant differences were identified between ER-low and ER-negative patients [34].

Fei et al. analyzed the clinicopathological characteristics and survival outcomes of ER-low BC in a cohort of 4179 patients diagnosed between 1998 and 2018. Among these, ER-high, ER-low, and ER-negative tumors accounted for 2982 (71.4%), 97 (2.3%), and 1100 (26.3%) cases, respectively. Patients with ER-low disease were significantly younger (mean age 56.4 vs. 59.1 years, *p* = 0.039) and were more frequently African American (77.4% vs. 61.9%, *p* = 0.001). ER-low BC more commonly exhibited a ductal histology (83.5% vs. 71.4%, *p* = 0.005), higher histologic grade (grade II/III: 94.8% vs. 74.0%, *p* < 0.001), and larger tumor size (mean diameter 27.6 mm vs. 21.7 mm, *p* = 0.003), and were more often diagnosed at advanced clinical stages (stage II–IV: 69.1% vs. 48.8%, *p* = 0.001). In addition, ER-low tumors were less likely to express PRs (84.0% vs. 38.1%, *p* < 0.0001) and more likely to be HER2-positive (28.9% vs. 11.2%, *p* < 0.0001). Overall, ER-low BC demonstrated clinicopathological features comparable to ER-negative disease but was associated with a significantly better prognosis. Within the PR-positive subgroup, ER-low tumors showed worse survival outcomes compared with ER-high tumors, while demonstrating a trend toward improved survival relative to ER-negative disease. Conversely, PR-negative, ER-positive and ER-low tumors exhibited similar survival outcomes, both significantly superior to those observed in ER-negative BC [27].

On the other hand, a systematic Review and meta-analysis by Chen et al. showed that ER-low BC patients tended to have smaller (*p* = 0.0018) and well differentiated tumors (*p* < 0.0001) compared with ER-negative BC and improved survival [23].

A further study in three large cohorts of BC patients, the authors evaluated the correlation between ER expression levels and tumor characteristics and prognosis. A total of 65/1955 (3.3%) were ER-low BC. Globally, the highest proportion was identified among luminal B HER2 3+ (9.4%) and grade 3 (4.3%) tumors.

The risk of death from BC was lower in ER-low compared to ER-negative patients. Women diagnosed in 1995 or later, when adjuvant treatment was available, had a higher proportion of ER-low BC and their tumors were smaller, and had a lower grade and lower ki67 compared to patients diagnosed before 1995. No significant difference with respect to ER-high BC was identified [10].

Even if available evidence agrees on the strict similarity between ER-low tumors and TNBC, further studies are needed to provide clarifications for the above-mentioned controversial results.

## 3. Treatment Strategies: Current Evidence on the Role of Endocrine Treatment, Chemotherapy and Immunotherapy

Current evidence focuses on wat might be the best treatment option in ER-low BC patients in the early and advanced disease setting. The most controversial question concerns the role of ET in this category of patients and the role of immunotherapy, due to the similarities observed between ER-low BC and TNBC.

### 3.1. Endocrine Therapy (ET)

ET is still a point of discussion in ER-low BC. Evidence is controversial even if most demonstrate limited efficacy (Table 1). More specifically, the point to assess is not just about “whether ET is effective,” but more importantly “for whom it is effective.” This uncertainty might be related to the heterogeneity of ER expression, the ER signaling pathway activity beyond mere protein expression and the occurrence of ER loss, and interactions with other pathways (e.g., growth factor receptor pathways). ER loss and ER signaling “dilution” in BC is multifactorial, involving genetic, epigenetic, signaling, post-transcriptional, post-translational, hypoxic, and BRCA1-related mechanisms. Understanding these pathways, especially in ER-low BC, would be a key resource to predict ET resistance [35]. Yau et al. described a gene expression signature associated with loss of ER function and oxidative stress in ER-positive BC, linking impaired ER function with a poorer prognosis and reduced ET responsiveness [36]. Evidence revealed that normal ER signaling is lost and tumor-specific ER signaling is gained during BC tumorigenesis. BC-specific ER cistromas showed an enrichment in DNA binding motif GRHL2, which demonstrated a crucial role for estrogen-stimulated proliferation of ERs + BC. Furthermore, its depletion led to an altered ER binding and differential transcriptional responses to estrogen stimulation. DLC1, an estrogen-induced tumor suppressor in the normal mammary gland, was found to have decreased expression in BC. In clinical cohorts, loss of DLC1 and gain of GRHL2 expression were associated with ER-positive BC and were independently predictive for worse survival [37]. A recent study found that activated glucocorticoid receptor is able to silence ER signaling by downregulating ESR1 expression and eliminating ER binding to regulatory elements, providing a mechanism of functional ER loss with implications for resistance [38].

Preclinical evidence showed that tamoxifen reduced epithelial cell volume in ER-positive tumors but not in ER-low BC [39].

Cheng et al. conducted a systematic review and metanalysis to compare survival outcomes of ER-low BC patients (1–9%) to ER-high in order to assess ET response and prognosis in ER-low BC [23]. After revision of inclusion and exclusion criteria, six retrospective studies were eligible for this study. Four studies performed survival comparisons among BC patients with different ER expression [34,41,42,43], whereas two studies assessed the optimal cut-off for defining ER positivity by analyzing survival differences using different cutoffs [44,45]. No significant differences were identified in the 5-year disease-free survival (DFS) among ER-low and ER-negative patients receiving ET (pooled OR, 1.51; ER-low versus ER-negative *p* = 0.145). Conversely, ER-high BC patients showed a significantly better 5-year DFS than ER-low (pooled OR, 0.52; ER-low versus ER-high; *p* = 0.034). When assessing survival in ER-low patients between those receiving ET and those not treated with ET, no significant difference in 5-year overall survival (OS) rate was reported (pooled OR, 0.87, with versus without ET; *p* = 0.684), as well as for 5-year relapse-free survival (RFS) rate (86.5% with ET versus 84% without ET; *p* = 0.679). Thus, this study failed to show a significant survival benefit from ET in ER-low patients [23].

In a study assessing 1824 consecutive cases of BC diagnosed between 2002 and 2009, for women with available data on treatment received, 66% were ER-low. For those treated with ET, a significantly worse outcome than the ER-high cancer patients was reported; conversely, no difference from ER-negative patients was observed (without ET) [32].

In a retrospective study by Yi et al. based on 9639 BC patients, ER-low received adjuvant chemotherapy more frequently (49.2% versus 35.5%; *p* < 0.0001) and adjuvant ET (20.4% versus 83.6%, *p* < 0.0001) less frequently than patients with ER-high tumors; on the contrary they were more likely to receive adjuvant ET when compared with ER-negative patients (20.4% versus 12.9%, *p* = 0.002). As for recurrences, ER-low patients treated with ET experienced higher rates than ER-high (17.7% versus 7.7%, *p* = 0.02) but no significant difference in total recurrences between these groups for patients not receiving ET were observed. Patients with ER-low BC had worse distant RFS (*p* = 0.0003), RFS (*p* = 0.0005) and OS (*p* < 0.0001) rates compared to ER-high ones, even if treated with ET. No differences were observed between ER-low and ER-negative patients not receiving ET. Thus, this study supports evidence in the absence of a clear benefit of ET in ER-low BC [34].

Ovarian function suppression (OFS) in premenopausal hormone receptor-positive BC may also be recommended in association to exemestane rather than tamoxifen in patients with a higher recurrence risk, as demonstrated by the TEXT and SOFT trials [46,47,48]. However, data on OFS in ER-low BC patients are lacking, since in these trials ER/PR expression had to be at least 10% of the cells and in the enrolled population, 95% had ER expression more than 50%. Thus, available evidence on OFS is insufficient for it to be already recommended in premenopausal ER-low patients, and further research is needed [11].

When Fei et al. compared the clinical outcomes of ER-low-positive/PR-positive, ET was associated with a better RFS (hazard ratio [HR] 0.111 (0.015–0.800), *p* = 0.03) and disease-specific survival (HR 0.093 [0.012–0.696], *p* = 0.02), even if a higher level of ERs was found in those receiving ET (mean 9%, range 5–10% versus mean 5%, range 1–10%; *p* = 0.0001). Conversely, when comparing the clinical outcomes of ER-low-positive/PR-negative tumors treated or not with ET, no significant difference was identified in RFS [HR 0.408 (0.037–2.657), *p* = 0.3] or disease-specific survival [HR 0.437 (0.045–2.998), *p* = 0.4]. According to the authors, these findings might support the eligibility of ER-low BC for ET [27].

Choong et al. evaluated the benefit of ET in early-stage ER-low BC who received chemotherapy, along with the frequency of ET omission and its association with OS in a retrospective cohort study by using the National Cancer Database. Patients coded as treated with ET and with a timing of ET start (before/after definitive surgery) were considered as having received adjuvant ET from that point onward. Stop times of ET and consequently treatment duration were not available, as well as data on the specific ET agents and compliance [13]. In ER-low BC, ET omission was significantly associated with worse OS in patients with tumors with ER expression between 6 and 10% (HR, 1.42 [95% confidence interval—CI: 1.00–2.02]; *p* = 0.048) but not ERs between 1 and 5% (HR 1.15 [95% CI: 0.91–1.45]; *p* = 0.24). In an exploratory analysis, ET omission in ER-low patients treated with adjuvant chemotherapy and in those with a pathological complete response (pCR) after neoadjuvant chemotherapy was not significantly associated with OS (HR 1.06 [95% CI: 0.62–1.80]; *p* = 0.84). Conversely, in ER-low patients with residual disease after neoadjuvant chemotherapy, ET omission was significantly related to shorter OS (HR 1.26 [95% CI: 1.00–1.57]; *p* = 0.046).

Thus, according to this study, since ET omission in ER-low, early-stage BC, is associated with significantly worse survival, patients with ER-low BC should not be automatically excluded from offers of ET until prospective data are available [13].

A prospective cohort study of 10.696 BC patients diagnosed in China between 2010 and 2020 assessed the association between ET and BC-specific survival in specific groups with respect to the regimen and the duration of ET.

Patients were classified into three groups based on the regimen and duration of ET. The regimens included aromatase inhibitor monotherapy or sequential tamoxifen followed by an aromatase inhibitor (aromatase inhibitor/tamoxifen + aromatase inhibitor), or only tamoxifen and no ET; the duration of ET included 2–3 years and >3 years. A total of 407 BC patients were ER-low. In this subset of patients, ET administration was associated with improved survival, especially with regimens including an aromatase inhibitor/tamoxifen + aromatase inhibitor compared to patients without ET. Moreover, patients receiving more than 3 years of ET showed an improvement in DFS, whereas there was no significant difference in BC-specific survival between patients with 2–3 years and >3 years of ET. The authors conclude that ET with an aromatase inhibitor/tamoxifen + aromatase inhibitor may be a reasonable treatment option, even if it requires validation in randomized studies [40].

Since definitive data on the application of ET in ER-low BC patients are lacking, further studies are required to assess the role of ET in this subset of BC patients. The limited benefit of ET should be carefully weighed against the potential adverse events and other options should also be taken in consideration [49]. Surely, it would be helpful if ER cut-off levels would be further investigated with the aim to personalize ET in ER-low BC.

In summary, current evidence indicates that, for the vast majority of ER-low patients (especially those who are PR-negative), ET offers limited and uncertain benefits; however, for patients with ER expression close to 10% (e.g., 6–10%), PR-positive status, or those who do not achieve pCR after chemotherapy, there may still be some benefit, and thus ET should not be automatically excluded and might be considered a reasonable option.

### 3.2. Chemotherapy

Yoder et al. performed a study based on a prospective multisite registry of 516 patients treated in the contemporary era to assess the impact of ER/PR-low versus ER/PR-negative on clinicopathologic characteristics, treatment patterns, and survival in HER2-negative BC patients. A total of 87.4% of patients had TNBC and 12.6% had ER-low BC. Globally, 97.7% of patients were treated with chemotherapy, with similar regimens; 97.8% were ER-low and 96.9% TNBC. A lower proportion of ER-low patients received neoadjuvant versus adjuvant chemotherapy when compared to TNBC (60.0% versus 70.7%, *p* = 0.079); similar pCR rates were reported between ER-low and TNBC (51.3% and 49.2 as observed in 49.2%, respectively (*p* = 0.808). Adjuvant chemotherapy was administered in 20% of ER-low patients. No significant difference in RFS and OS between ER-low and TNBC was detected (3-year RFS 82.4% and 82.5% among ER-low and TNBC, respectively; HR 0.90; 95% CI: 0.50–1.62, *p* = 0.728; 3-year OS: 83.4% versus 88%, HR 0.85; 95% CI: 0.44–1.66, *p* = 0.632). As for patients receiving neoadjuvant chemotherapy, pCR was related to significantly better RFS and OS compared to patients without pCR and the results were similar in both ER-low and TNBC patients (3-year 100% and 94.3% in TNBC; 3-year OS 100% versus 97.7%, respectively). Among patients with residual disease, RFS and OS were similar in the ER-low and TNBC groups (3-year RFS 68.4% in ER-low versus 67.6% in TNBC, HR 0.88, 95% CI: 0.40–1.93, *p* = 0.738; 3-year OS and 68% versus 76.9%, HR 0.77, 95% CI: 0.32–1.81, *p* = 0.540) [14].

Dieci et al. assessed clinical outcomes of ER-low BC versus ER-negative mono-institutional cohort of non-metastatic BC patients receiving (neo)adjuvant chemotherapy. pCR rates were similar ER-low and ER-negative patients treated with neoadjuvant chemotherapy (44% ER-low and 38% ER-negative, *p* = 0.498). In both groups, pCR was related to long-term outcome (5-year invasive RFS 100% for pCR versus 50% for residual disease in the ER-low group, 89.5% for pCR versus 51.6% for residual disease in the ER-negative group) and relapses occurred early, mostly in the first 3 years after diagnosis. No ER-low BC patients achieving pCR were treated with adjuvant ET. Thus, early ER-low BC showed a similar response to neoadjuvant chemotherapy to TNBC [21].

### 3.3. Immunotherapy in Combination with Chemotherapy

Due to the similarities observed between ER-low BC and TNBC, there is evidence that immune-checkpoint inhibitors (ICIs) in combination with chemotherapy might have meaningful clinical activity in ER-low patients in the neoadjuvant setting. Several aspects can influence response to ICIs. Even if no prospectively validated predictive factors are available, active research focuses on the tumor microenvironment (TME), biomarker expression, and the potential role of dysregulated lipid metabolism in modulating immune cell function and inflammatory responses [50].

The KEYNOTE-522 established a new standard of treatment for early-stage TNBC with the addition of pembrolizumab to chemotherapy in the neoadjuvant and adjuvant setting for TNBC, since the combination with immunotherapy significantly improved pCR, event-free survival and OS. However, ER-low patients were not included in this trial [51]. Then, other trials assessed the role of ICIs in ER-positive BC and included a limited number of ER-low patients.

The KEYNOTE-756 trial included 1278 grade 3, stage II-III ER-positive/HER2-negative BC patients who received pembrolizumab or placebo plus taxane and anthracycline-based chemotherapy; among them, only 77 were ER-low (34 in the pembrolizumab arm and 43 in the control arm) [52].

The CheckMate 7FL trial enrolled 510 high-risk ER-positive stage II-III BC patients, including grade 3 ER ≥ 1% and grade 2 ER 1–10% who were randomized to receive standard neoadjuvant anthracycline and taxane-based chemotherapy plus nivolumab or placebo. Among them, 32 were ER-low (18 in the nivolumab arm and 14 in the control arm). The addition of nivolumab significantly increased the pCR rate; more specifically, pCR and residual cancer burden (RCB) 0 or I rates were higher for patients with lower ER (<50%) and/or progesterone receptor expression (<10%) BC than in patients with higher ER or PR expression [53].

“PROMENADE” is a French, retrospective real-world study conducted in 16 comprehensive cancer centers. Data from ER-low HER2-negative BC patients treated with KEYNOTE-522 regimen since 2022 were collected. The primary objective was the locally assessed pCR rate. At total of 155 patients were included; median age was 47.6 years (range 19.5–80.1 years), and 57.4% were premenopausal. Overall, 88.4% had a tumor size ≥T2; 53.5% were node-negative. Most BC had aggressive features: 85.6% were grade 3 and a median Ki67 was 70%. A total of 51.6% had HER2-low BC. After surgery, a high rate of patients achieved a pCR (71.9%), which was significantly related to grade 3 and stage III in the multivariable analysis. These findings seem to reinforce the idea that ER-low HER2-negative BC should be treated in the neoadjuvant setting as TNBC to maximize pCR [54].

Colombo Bonadio et al. conducted an analysis as part of the Neo-Real/GBECAM-0123 study, a multicentric, real-world data initiative across ten Brazilian cancer centers in order to evaluate the outcomes of neoadjuvant pembrolizumab plus chemotherapy in ER-low BC patients [16]. The primary objective was to assess the effectiveness of neoadjuvant pembrolizumab in combination with chemotherapy in the ER-low population; primary endpoints included pCR and RCB. Among 410 patients included in the Neo-Real study (from June 2020 to June 2024), 20 patients had an ER-low/HER2-negative BC treated with neoadjuvant pembrolizumab plus chemotherapy. They were mainly young patients (median age 40 years—range 28–64); 90% had grade 3 BC and a high Ki67 index (median 75%—range 30–95%) and 70% had a stage II BC. Most patients (85%) had ER-low expression and negative PR expression. The pCR rate was 60% (12/20) and RCB 0–1 rate was 65%. In the univariate logistic regression analysis, the only variable showing a trend toward a lower pCR rate was the administration of fewer than six cycles of neoadjuvant pembrolizumab. Regarding safety, grade ≥3 adverse events were reported in 30% of patients, with grade ≥3 neutropenia being the most frequent (20%). One patient (5%) experienced a grade ≥3 immune-related hypophysitis, and 10% of patients discontinued treatment due to toxicity. Overall, these findings were consistent with those observed in the entire Neo-Real cohort. Moreover, these findings underline the similar response of ER-low tumors to that of ER-negative BC in terms of pCR rates in the global cohort of the Neo-Real study and in prospective clinical trials. Furthermore, the pCR and RCB 0–1 rates were similar to ER-low BC patients enrolled in the Keynote 756, CheckMate 7FL and PROMENADE study. According to these results, the Authors conclude that the management of ER-low BC patients should be the same as TNBC [16].

Da Silva et al. conducted a systematic review and meta-analysis to assess the efficacy and safety of neoadjuvant chemoimmunotherapy in patients with ER-low/HER2-negative BC. The primary endpoint was the pCR rate, while a secondary descriptive analysis focused on safety outcomes. Seven studies, comprising a total of 260 patients with ER-low BC (three cohort studies and four clinical trials), were included. Among these, four studies evaluated pembrolizumab, one nivolumab, and one camrelizumab. The pooled pCR rate for ER-low BC was 64.88% (95% CI: 56.72–73.04; I^2^ = 37.5%), with no significant difference observed between clinical trials and cohort studies (*p* = 0.724). Safety data were available in two studies and suggested a slightly lower incidence of severe adverse events, although the limited sample size precluded definitive conclusions. Overall, this meta-analysis confirmed high pCR rates in ER-low BC treated with chemoimmunotherapy, comparable to those reported in TNBC [55].

Table 2 shows the main studies with neoadjuvant chemotherapy plus an ICI that included ER-low BC patients.

## 4. Artificial Intelligence Application in ER-Low BC Pathology

The introduction of machine learning and deep learning (DL) systems in digital pathology has deeply impacted the field of cancer diagnosis, aiding histopathologists to perform more accurate and efficient pathological diagnoses [56]. AI platforms have made significant strides in BC diagnosis and prognosis, allowing a marked advancement in digital pathology of BC towards precision medicine [57]. The development of AI models focused on BC diagnosis, such as Visiopharm ER, Path AI, Mindpeak Breast, Ibex Galen Breast, Paige Breast Suite and Aiforia have represented a breakthrough in this complex field of oncology, with a relevant impact on BC pathology [58]. Each of these platforms has been developed with a peculiar objective. PathAI can assist pathologists in the identification and classification of various types of BC, including in situ, minimally invasive and invasive ductal adenocarcinoma, with high precision. Ibex Galen Breast is focused on BC cell detection in whole slide images of needle biopsies, providing pathologists with subtle features that might be missed during conventional breast cancer sample analysis at the microscope [59]. The CE-IVD marked Aiforia^®^ Clinical AI Model for Breast Cancer; ER. Indeed, this tool performs the calculation of ER-positive and ER-negative cells from whole slide images (WSIs) and selected areas; moreover, it is able to view and select areas with high ER-positive density or hotspots [60].

Among the AI-driven models developed to assist pathologists in BC diagnosis, the Visiopharm ER BC AI is of particular interest. This model provides the total number of nuclei of cancer cells in the tissue sample, and the number of positive nuclei. Additionally, the algorithm gives information regarding the intensity of the staining of immunoreactive nuclei. The AI model works fully automated in an integrated setting, starting from detecting invasive cancer cells and separating them from normal breast gland components and necrosis. The final step includes the detection of nuclei and their classification, ending with the export of the relevant outputs about the quantification of the ER-expressing nuclei. The outputs of the model are quantification of total nuclei, percentage of ER-positive nuclei and the Alfred score, which takes into consideration both the proportion score and the intensity score, reflecting the intensity of the ER-positive nuclei. The automated digital imaging analysis of ER immunohistochemistry has shown excellent concordance with pathologists’ scores and has been proposed as a new tool in the clinical setting, being able to save time and labor for pathologists [61].

A more recent pilot study aimed to verify the performance of the Visiopharm AI algorithm for ERs in cytology specimens from BC metastases confirmed the excellent concordance of the algorithm with pathologists’ assessments, but evidenced that precision in identifying tumor cells in cytology specimens requires further enhancement [62]. Studies on the application of AI-driven models to ER status determination on cytological specimens induced Lossos C. and coworkers to evaluate the value of immunostains of cell blocks and core needle biopsy specimens of metastatic BC [63]. The authors, on the basis of their experience, suggested that classical IHC on a cell block or fine needle biopsy can provide reliable information on the ER status of BC.

The relevant role of AI models in improving pathologists’ concordance in the classification of ER status in BC has been underlined by a recent study that underscored the significant role of AI analyzers in improving pathologists’ concordance in the BC classification of molecular subtypes [64]. In this study, when compared to initial interpretations on the ER status performed by three board-certified pathologists, the assistance of AI led to an increase in the agreement among pathologists, from 93% to 96.5%.

Interesting data emerged from a very recent study focused on a DL model, PANProfiler Breast, which was developed to identify ER status through the technique of virtual immunostaining [65]. This new AI-driven model showed high concordance for ER status identified from whole slide images of routinely stained Hematoxylin and Eosin (H&E) BC samples and the corresponding pathology reports based on classical IHC, with a concordance across the cohorts of 90–93%. This study confirmed a previous study that some years ago indicated that image analysis with DL methods can predict IHC marker expression, including ER status, in BC based solely on H&E digitalized images [66].

These data taken together clearly show the potential of AI-driven models for the identification of the ER status in BC, with a high concordance between AI-supported findings and the evaluation of expert pathologists. Unfortunately, expert pathologists dedicated exclusively to breast pathology can be encountered exclusively in large pathology departments, in which super-specialization of pathologists can be programmed. Different situations occur in small peripheral hospitals and in low-income countries, in which general pathologists analyze biopsies from every district of the body. In these settings, DL algorithms could significantly improve the pathologists’ ability in the detection of the ER status, increasing the significance of the pathology review of metastatic BC [66,67,68].

Utilizing another DL algorithm, Shamai and coworkers showed the ability of their AI model to predict ER status with an accuracy of 92% [69]. The more recent employment of foundational models such as ResNet50 and Transformer showed the ability of AI models to provide more precise clinical treatment and prognostic evaluations for BC patients [70].

## 5. Future Perspectives

Multimodal AI refers to a machine learning system able to integrate diverse data types, including medical images (WSI of histology, radiology imaging) clinical data, and genomic data (DNA sequencing) to enhance decision-making in health care [71]. Thus, multimodal AI represents the next-generation technology beyond the single task of AI models previously described (for instance, ER expression assessment). By integrating multidimensional data including histology, genomics and imaging, it shows potential for more precise subtyping and treatment prediction for ER-low BC patients. Indeed, emerging multimodal AI models are reshaping the field of human pathology, offering new solutions to enhance diagnostic accuracy and support clinical decision. Furthermore, these new tools contribute significantly to biomarker discovery in tumor cells, tailoring treatment plans and therapies based on individual genetic and biomarker profiles [72]. Furthermore, multimodal DL systems allow the integrative histology–genomic–biomarker analysis of multiple cancer types [73]. The most novel AI models, such as ResoMergeNet, represent a substantial advancement in diagnostic and prognostication accuracy for BC, reducing diagnostic errors often due to the inter-observer variability in marker expression evaluation, such as ER status, eventually enhancing BC diagnosis and patient outcomes [74].

Risk stratification remains a critical challenge in BC patients for optimal diagnosis and therapy selection. This assumption fits very well with ER-low BC patients: some of them show strict similarities with ER-negative patients, and are not responsive to ET. Other ER-low patients show similarities with ER-positive patients, who are responsive to ET. AI-powered models could play a relevant role in the stratification of ER-low patients, allowing their early distinction and the early correct therapy in the subgroup of ER-low BC patients who are responsive to ET. The way to reach this goal comes from a recent study by Schallemberg and coworkers, who analyzed the TME in lung cancer, with the aim of stratifying patients with the same cancer histotype and same stage [75]. The AI model analyzed the complex cellular relationships between cancer cells, lymphocytes, tumor-associated monocytes (TAMs) and cancer-associated fibroblasts (CAFs) of the TME, identifying different types of cell niches: hot niches, rich in inflammatory cells and cold niches, devoid of the inflammatory component. A high concentration of hot niches had a predictive power, being associated with a poor prognosis and lower survival. TME has indeed shown to be crucial also in metastatic spread in BC and it presents with different microenvironmental characteristics in different organs, underlying the pivotal role of immune and resident cells in the development of distinct metastatic foci [76].

The application of a similar approach to the AI-powered analysis of BC samples could allow a stratification of low-ER BC patients, allowing the identification of patients who are possibly responsive to ET.

AI tools, and in particular multimodal AI models, thanks to their diagnostic accuracy in quantifying ER expression, might resolve uncertainty in ER-low BC classification and consequently in risk stratification, which is crucial to select the most appropriate treatment strategy for these patients. The ability of AI to precisely identify ER-positive cells by integration of several data types and to improve agreement among pathologists and facilitate their work is a promising instrument with significant implications in practical clinical decision-making. For instance, it might be useful to identify which patients are more likely to benefit from ET, where evidence is still uncertain.

Currently, various clinical trials are assessing the benefit of new treatment strategies in ER-low BC.

NCT02115048 is an open-label, randomized phase II trial assessing the role in terms of PFS of first-line afatinib, a highly selective, irreversible tyrosine kinase receptor inhibitor of HER2, and epidermal growth factor receptor (EGFR) in combination with conventional ET (letrozole) versus letrozole alone in a population of advanced ER-low, HER2-negative post-menopausal BC patients. The rationale for testing the combination of EGFR inhibition and anti-estrogen agents is based on the activation of MAPK and PI3K/AKT signaling and crosstalk between ER and growth factor pathways as potential mechanisms of ER downregulation. More specifically, the crosstalk between steroid hormone signaling and peptide growth factor signaling through the HER-family, as well as the relationship between peptide growth factor signaling and hormone receptor status has been described in BC [77,78]. Furthermore, EGFR expression and HER2 amplification have been demonstrated to influence resistance to anti-estrogen treatment in laboratory models and clinical studies [79,80,81]. Thus, the association of these treatment strategies might improve the PFS of this subgroup of BC.

Neoadjuvant olaparib plus durvalumab in ER-low BC is under evaluation in an ongoing trial in the BRCAness subgroup of ER-low BC (NCT03594396). Olaparib is a poly (ADP-ribose) polymerase (PARP) inhibitor that has been proven to be effective not only in germline BRCA1/2 mutant BC patients but also in tumors with deficient homologous recombination DNA repair (BRCAness patients), as sometimes occurs in TNBC and ER-low BC. The trial rationale is based also on the potential synergy between PARP inhibitor and ICIs (as durvalumab) due to an increased mutation burden, neoantigen expression and immunogenic cell death; this combination might induce an increased pCR rate in the neoadjuvant setting [82].

As for CDK4/6 inhibitors, their role in ER-low BC has not yet been established, thus, their use in this category of patients should be cautious [11]. Indeed, current data are lacking and cannot allow a full understanding of their impact. As for the MONALEESA trials, secondary analysis showed that ribociclib improved PFS across all subgroups except the basal-like subtype, of which ER-low tumors often present with typical features [83]. For the MonarchE and Natalee trial, the inclusion criteria were consistent with the ASCO/CAP guidelines and in hormone receptor-positive tumors with ER expression ≥1% could be enrolled [84,85]. A secondary analysis of the monarchE trial assessing the efficacy of abemaciclib according to ER expression levels included only ER expression starting from 10%, excluding the “real” ER-low population [83,86]. Goetz et al. performed a STEPP analysis of several different windows of ER expression and observed similar HR for invasive DFS comparing ER 1–10% (HR, 0.842 [95% CI, 0.384–1.846]) with ER 11–30% (HR, 0.896 [95% CI, 0.458–1.752]; interaction *p* = 0.9065). As for the potential lack of benefit of CDK 4/6 inhibitors in the basal subtype, a secondary biomarker analysis of the monarchE trial evaluating the prognostic and predictive role of intrinsic subgroups revealed a consistent invasive DFS treatment effect across all intrinsic molecular subtypes, including the basal one (interaction *p* value for all subtypes = 0.621) [87].

These data suggest that if there is a functional ER pathway, it can be supposed that CDK4/6 inhibitors in combination with ET may be effective. Thus, further research is needed to define definitive conclusions on their role in ER-low BC.

Additional strategies are being evaluated to improve outcomes for BC, including those in both the ER-positive and TNBC subgroups. Their role in the specific ER-low BC population remains to be investigated but could represent an important avenue for future research in these patients.

Physical activity has been reported to be associated with reduced recurrence and mortality in BC patients and offers benefits across prevention, treatment, and survivorship. Evidence suggests that exercise can modulate biological mechanisms that enhance therapy effectiveness, and higher activity levels during chemotherapy, radiotherapy, or surgery; moreover, it improves overall health and treatment outcomes [88].

Photodynamic therapy in BC treatment is limited by inadequate immunogenicity and inefficient pyroptosis induction. Dissolvable microneedles for localized co-delivery of decitabine and glutathione-responsive photosensitizer nanoparticles to enhance immunogenic pyroptosis in BC were developed and showed pyroptosis induction together with the promotion of dendritic cells and CD8+ T cells responses. In vivo, this approach suppressed primary tumors and, combined with anti-PD-1, prevented recurrence and metastasis, establishing durable systemic immunity [89].

Myeloid-derived suppressor cells (MDSCs) promote migration and invasion of ER+ BC cells via exosomal miR-155-5p, which downregulates SIRT1 and drives epithelial–mesenchymal transition (EMT). TME-responsive polymeric micelles co-delivering alpelisib (a PI3K inhibitor) and cobomarsen (a miR-155-5p inhibitor) were developed by Chen et al., achieving targeted delivery and intracellular stability. This dual-targeting strategy suppressed PIK3CA-mutant tumor growth and EMT, highlighting a novel nanotherapeutic approach against MDSC-driven metastasis in ER-positive BC [90].

Huang et al. designed a water-soluble nanocarrier, PEI-GCP(Z)/mPEG, to co-deliver paclitaxel (PTX) and the photosensitizer IR783, forming nanoparticles with high drug-loading efficiency. It enabled combined chemotherapy and photothermal therapy under 808 nm laser irradiation, while also inducing immunogenic cell death, promoting dendritic cell maturation, and enhancing cytotoxic and memory T cell responses. In vitro and in vivo, it effectively targeted tumors and significantly inhibited growth, demonstrating its potential as a multimodal therapy for TNBC [91].

## 6. Conclusions

ER-low BC is increasingly recognized as a distinct and biologically heterogeneous entity that challenges the traditional dichotomy between hormone receptor-positive BC and TNBC. Despite its historical classification as ER-positive, growing pathological, molecular, and clinical evidence shows that ER-low BC often behaves more similarly to ER-negative BC, with limited and inconsistent benefit from ET and a treatment response profile that is more likely for TNBC, especially in the neoadjuvant chemo-immunotherapy setting. At the same time, a small subset of ER-low tumors may retain endocrine sensitivity, underscoring the need for more accurate stratification tools.

**Figure 2:**
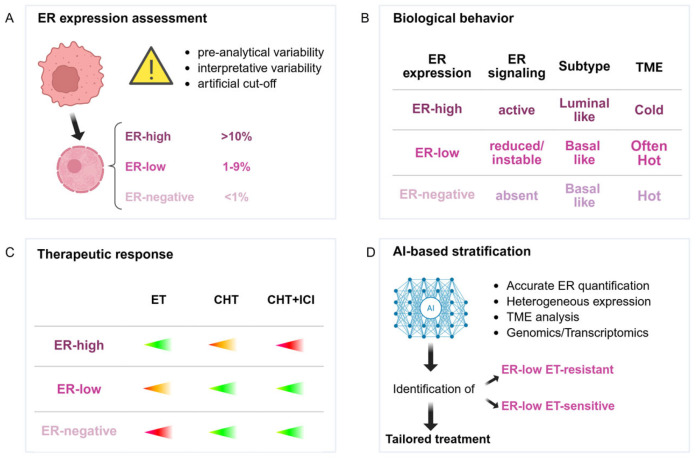
From ER expression to therapeutic decision in ER-low BC. (**A**) ER expression assessment; (**B**) biological behavior; (**C**) therapeutic response; (**D**) AI-based stratification. Abbreviations: AI: artificial intelligence; CHT: chemotherapy; ER: estrogen receptor; ET: endocrine treatment; ICIs: immune-checkpoint inhibitors; TME: tumor microenvironment. The figure was created with www.biorender.com, and the appropriate license for publication was obtained.

Advances in AI, including digital pathology platforms and emerging multimodal models, represent a promising avenue to refine ER quantification, reduce diagnostic variability, and ultimately identify which ER-low patients may truly benefit from ET, in order to maximize treatment efficacy for this subgroup (Figure 2). Future prospective trials and AI-driven approaches will be essential to clarify optimal treatment strategies, improve patient selection, and personalize therapy in this challenging and evolving BC subtype.

## Figures and Tables

**Table 1:** Studies assessing the role of endocrine treatment in ER-low BC.

Studies Demonstrating ET Inefficacy	Studies Demonstrating ET Efficacy
Leeper, A.D. et al. [39]: no significant change in tumor volume between untreated and tamoxifen treated preparations	Fei et al. [27]: improved RFS and disease-specific survival in PR-positive; no significant difference in RFS or disease-specific survival in PR-negative disease (role of PR?)
Chen, T. et al. [23]: no significant difference in 5-year OS rate and 5-year RFS rate by ET treatment	Choong et al. [13]: worse OS without ET in case of ER expression 6–10% and in patients with residual disease after neoadjuvant CHT
Poon et al. [32]: significantly worse outcome with ET compared to ER-high	Xie et al. [40]: improved survival; improved DFS in patients receiving ET for more than 3 years
Yi et al. [34]: no significant differences in total recurrences between patients receiving or not receiving ET; worse distant RFS, RFS and OS rates irrespective of ET	-

CHT: chemotherapy; DFS: disease-free survival; ET: endocrine treatment; OS: overall survival; PR: progesterone receptors; RFS: relapse free survival.

**Table 2:** Neoadjuvant studies assessing immunotherapy plus chemotherapy that included ER-low BC patients.

Study Name/Author	Study Type	Total Number of BC Patients	Number of ER-Low Patients	Treatment Strategies
KEYNOTE-756 [52]	randomized, double-blind, placebo-controlled phase 3 study	1278	77 (34 in experimental arm and 43 in the control arm)	pembrolizumab or placebo + anthracycline and taxane-based chemotherapy
CHECKMATE 7FL [53]	randomized, double-blind, placebo-controlled phase 3 study	510	32 (18 in the experimental arm and 14 in the control arm)	nivolumab or placebo + anthracycline and taxane-based chemotherapy
PROMENADE [54]	multicentric, retrospective real-world study	155	155	pembrolizumab + anthracycline and taxane-based chemotherapy
Neo Real—GBECAM0123 [16]	multicentric, real-world study	410	20	pembrolizumab + anthracycline and taxane-based chemotherapy
Da Silva [55]	systematic review and meta-analysis	260	260	Pembrolizumab or nivolumab or camrelizumab + anthracycline and taxane-based chemotherapy

## Data Availability

The data presented in this study are available in this article.

## Abbreviations

The following abbreviations are used in this manuscript:

AI	Artificial intelligence
ASCO	American Society of Clinical Oncology
ASCO/CAP	American Society of Clinical Oncology/College of American Pathologists
BRCA	Breast Cancer genes
BC	Breast cancer
CI	Confidence interval
DFS	Disease-free survival
DL	Deep learning
EGFR	Epidermal growth factor receptor
EMT	Epithelial–mesenchymal transition
ER	Estrogen receptor
ET	Endocrine treatment
H&E	Hematoxylin and eosin
HER2	Human epidermal growth factor receptor
HR	Hazard ratio
ICI	Immune-checkpoint inhibitors
IHC	Immunohistochemistry
MDSC	Myeloid-derived suppressor cells
OFS	Ovarian function suppression
OS	Overall survival
PARP	Poly (ADP-ribose) polymerase
pCR	Pathological complete response
PR	Progesterone receptor
RCB	Residual cancer burden
RFS	Relapse free survival
TME	Tumor microenvironment
TNBC	Triple-negative breast cancer
WSI	Whole slide images
